# Supplementary material for: A parsimonious model of blood glucose homeostasis
Source: PLOS Digit Health. 2022 Jul 14;1(7):e0000072. doi: 10.1371/journal.pdig.0000072 (PMC9931355; doi:10.1371/journal.pdig.0000072)
Supplement: S1 Thm — On the stability of stationary points of the glucose homeostasis control model. Proof. Under the assumption of a constant input, consider first that the input is greater than the resting metabolic rate, i.e. F > A3, then G > 0. Therefore the stationary point corresponding to e* < 0 no longer exists. If the solution is of class C2(D), linearizing around (u*, e*) to get the Jacobian matrix J=[-λ+A1∂f∂u|(u*,e*)λ(A1+A2)+A1∂f∂e|(u*,e*)∂f∂u|(u*,e*)∂f∂e|(u*,e*)] (15) where 2∂f∂u=-(e*+e¯),∂f∂e=-u*. (16) Thus trJ=-λ-A1(e*+e¯)-u*<0detJ=λu*+λ(A1+A2)(e*+e¯)>0 Therefore the stationary point is a stable node or focus for G > 0. If G < 0, the linearization has the properties trJ=-λ-A1e¯<0 and detJ=λe¯(A1+A2)>0 which yields a stable node or focus. Hence the stationary point (u*, e*) is always asymptotically stable. (PDF) [file pdig.0000072.s001.pdf]

S1 Thm. Proof of Theorem. On the stability of stationary points of the glucose homeostasis control model.

Proof. Under the assumption of a constant input, consider first that the input is greater than the resting metabolic rate, i.e. $F > A_3$, then $G > 0$. Therefore the stationary point corresponding to $e^* < 0$ no longer exists. If the solution is of class $C^2(D)$, linearizing around (u^*, e^*) to get the Jacobian matrix

$$J = \begin{bmatrix} -\lambda + A_1 \frac{\partial f}{\partial u}|_{(u^*,e^*)} & \lambda(A_1 + A_2) + A_1 \frac{\partial f}{\partial e}|_{(u^*,e^*)} \\ \frac{\partial f}{\partial u}|_{(u^*,e^*)} & \frac{\partial f}{\partial e}|_{(u^*,e^*)} \end{bmatrix} \tag{1}$$

where

$$\frac{\partial f}{\partial u} = -(e^* + \bar{e}), \qquad \frac{\partial f}{\partial e} = -u^*. \tag{2}$$

Thus

$$\begin{aligned} \mathrm{tr}J &= -\lambda - A_1(e^* + \bar{e}) - u^* < 0 \\ \det J &= \lambda u^* + \lambda(A_1 + A_2)(e^* + \bar{e}) > 0 \end{aligned}$$

Therefore the stationary point is a stable node or focus for $G > 0$. If $G < 0$, the linearization has the properties $\mathrm{tr}J = -\lambda - A_1\bar{e} < 0$ and $\det J = \lambda\bar{e}(A_1 + A_2) > 0$ which yields a stable node or focus. Hence the stationary point (u^*, e^*) is always asymptotically stable. □
