# Supplementary material for: A parsimonious model of blood glucose homeostasis
Source: PLOS Digit Health. 2022 Jul 14;1(7):e0000072. doi: 10.1371/journal.pdig.0000072 (PMC9931355; doi:10.1371/journal.pdig.0000072)
Supplement: S2 Thm — On determining a trapping region under the assumption of a constant input. Proof. For ease of notation, we introduce the auxiliary variable v = u − A1 e. In the coordinate system (v, e), the ellipsoid implicitly defined by dL/dt = 0 has its major and minor axes aligned with the coordinate axes. It is enclosed by a rectangle with sides at v = vmin, vmax and e = emin, emax. A direct computation shows that vmin < -vmax < 0 < vmax. Since L is a monotonically increasing function of e on the domain D, the maximal value of L over the rectangle is then obtained at (vmin,emax-)=(-A2e¯2-12e¯A2A1A1A2e¯4+[A1e¯2+G]2,-e¯2+G2A1e¯+12A1e¯A1A2e¯4+[A1e¯2+G]2) if G≤-A2e¯2/4, in which case emax<0, and at (vmin,emax+)=(-A2e¯2-12e¯A2A1A1A2e¯4+[A1e¯2+G]2,-e¯2+12A1A12e¯2+A1A2e¯2+4A1G) if G>-A2e¯2/4, in which case emax>0. At this point we have L(vmin+A1emax±,emax±)=C± with C± as stated in the theorem. We now use the Bendixson-Dulac theorem to demonstrate that it is impossible for solutions that enter this region to be periodic, consider the equivalent dynamical system v˙=-λ(v+A2e)≡F1(v,e),e˙=-A3-(v+A1e)ϕ(e,e¯)≡F2(v,e). We now show that Q = ∂v F1 + ∂e F2 has the same sign in D. Computing Q directly, we get Q={-λ-A1(2e+e¯)-v,ife>0-λ-A1e¯,ife<0. It is clear that Q is constant and negative for any e < 0. Hence we only need to consider e > 0. Notice that Q is linear with respect to v and e, therefore any extrema of Q must be on the boundary of D. The maximal value can be found by the method of Lagrange multipliers, leading to Q(v*,e*)=-8A12+4A12e¯+4λA1+λA24A1<0 and so Q(v, e) < 0 in D. Therefore no periodic solution exists in the trapping region. Remark: Although the Bendixson-Dulac theorem is stated for differentiable vector fields, its property still holds for our model despite F2(v, e) being nondifferentiable along e = 0. (PDF) [file pdig.0000072.s002.pdf]

S2 Thm. **Proof of Theorem**. On determining a trapping region under the assumption of a constant input.

Proof. For ease of notation, we introduce the auxiliary variable $v = u - A_1 e$. In the coordinate system (v, e), the ellipsoid implicitly defined by $\mathrm{d}L/\mathrm{d}t = 0$ has its major and minor axes aligned with the coordinate axes. It is enclosed by a rectangle with sides at $v = v_{\min}$, $v_{\max}$ and $e = e_{\min}$, $e_{\max}$. A direct computation shows that $v_{\min} < -v_{\max} < 0 < v_{\max}$. Since L is a monotonically increasing function of e on the domain D, the maximal value of L over the rectangle is then obtained at

$$(v_{\min}, e^{-}_{\max}) = \Big(-\frac{A_2\bar{e}}{2} - \frac{1}{2\bar{e}}\sqrt{\frac{A_2}{A_1}}\sqrt{A_1A_2\bar{e}^4 + [A_1\bar{e}^2 + G]^2}, \\ -\frac{\bar{e}}{2} + \frac{G}{2A_1\bar{e}} + \frac{1}{2A_1\bar{e}}\sqrt{A_1A_2\bar{e}^4 + [A_1\bar{e}^2 + G]^2}\Big)$$

if $G \le -A_2\bar{e}^2/4$, in which case $e_{\max} < 0$, and at

$$(v_{\min}, e^{+}_{\max}) = \Big(-\frac{A_2\bar{e}}{2} - \frac{1}{2\bar{e}}\sqrt{\frac{A_2}{A_1}}\sqrt{A_1A_2\bar{e}^4 + [A_1\bar{e}^2 + G]^2}, \\ -\frac{\bar{e}}{2} + \frac{1}{2A_1}\sqrt{A_1^2\bar{e}^2 + A_1A_2\bar{e}^2 + 4A_1G}\Big)$$

if $G > -A_2\bar{e}^2/4$, in which case $e_{\max} > 0$. At this point we have $L(v_{\min} + A_1 e^{\pm}_{\max}, e^{\pm}_{\max}) = C_{\pm}$ with $C_{\pm}$ as stated in the theorem.

We now use the Bendixson-Dulac theorem to demonstrate that it is impossible for solutions that enter this region to be periodic, consider the equivalent dynamical system

$$\begin{aligned} \dot{v} &= -\lambda(v + A_2 e) \equiv F_1(v, e), \\ \dot{e} &= -A_3 - (v + A_1 e)\phi(e, \bar{e}) \equiv F_2(v, e). \end{aligned}$$

We now show that $Q = \partial_v F_1 + \partial_e F_2$ has the same sign in D. Computing Q directly, we get

$$Q = \begin{cases} -\lambda - A_1(2e + \bar{e}) - v, & \text{if } e > 0 \\ -\lambda - A_1\bar{e}, & \text{if } e < 0. \end{cases}$$

It is clear that Q is constant and negative for any $e < 0$. Hence we only need to consider $e > 0$. Notice that Q is linear with respect to v and e, therefore any extrema of Q must be on the boundary of D. The maximal value can be found by the method of Lagrange multipliers, leading to

$$Q(v^*, e^*) = -\frac{8A_1^2 + 4A_1^2\bar{e} + 4\lambda A_1 + \lambda A_2}{4A_1} < 0$$

and so $Q(v, e) < 0$ in D. Therefore no periodic solution exists in the trapping region. □

Remark: Although the Bendixson-Dulac theorem is stated for differentiable vector fields, its property still holds for our model despite $F_2(v, e)$ being nondifferentiable along $e = 0$.
