# Supplementary material for: A parsimonious model of blood glucose homeostasis
Source: PLOS Digit Health. 2022 Jul 14;1(7):e0000072. doi: 10.1371/journal.pdig.0000072 (PMC9931355; doi:10.1371/journal.pdig.0000072)
Supplement: S1 Cor — On determining a trapping region for any bounded input function. Proof. The curve L = C takes the form C = C− if G<-A2e¯2/4 and C = C+ if G≥-A2e¯2/4, and defines the boundary of a trapping region to Eqs 5 and 6. If Gmin≥-A2e¯2/4, then C = C+ everywhere in the domain. Notice that C+ is a strictly increasing function of G, therefore its maximum is C+(Gmax). Suppose now that Gmin<-A2e¯2/4. A direct computation of the second derivative of C− gives d2C-dG2=(2e¯2A1A2+4e¯λ)(A1A2e¯4[A1A2e¯4+(A1e¯2+G)2]32)+2>0 (17) with the inequality holding for all values of G. Therefore the maximum of C− occurs at one of its endpoints. Since C is continuous over all of G, and C+ is strictly increasing, then C-(-A2e¯2/4)<C+(Gmax), meaning that the right endpoint (relative to values of G(t)) of C− cannot be a maximum of C. Thus the largest value of C must be the larger of C−(Gmin) or C+(Gmax) as required. (PDF) [file pdig.0000072.s003.pdf]

S1 Cor. Proof of Corollary. On determining a trapping region for any bounded input function.

Proof. The curve $L = C$ takes the form $C = C_-$ if $G < -A_2\bar{e}^2/4$ and $C = C_+$ if $G \geq -A_2\bar{e}^2/4$, and defines the boundary of a trapping region to Eq **??**–**??**. If $G_{\min} \geq -A_2\bar{e}^2/4$, then $C = C_+$ everywhere in the domain. Notice that C_+ is a strictly increasing function of G, therefore its maximum is $C_+(G_{\max})$.

Suppose now that $G_{\min} < -A_2\bar{e}^2/4$. A direct computation of the second derivative of C_- gives

$$\frac{\mathrm{d}^2 C_-}{\mathrm{d}G^2} = (2\bar{e}^2\sqrt{A_1A_2} + 4\bar{e}\lambda)\left(\frac{A_1A_2\bar{e}^4}{[A_1A_2\bar{e}^4 + (A_1\bar{e}^2 + G)^2]^{\frac{3}{2}}}\right) + 2 > 0 \tag{3}$$

with the inequality holding for all values of G. Therefore the maximum of C_- occurs at one of its endpoints. Since C is continuous over all of G, and C_+ is strictly increasing, then $C_-(-A_2\bar{e}^2/4) < C_+(G_{\max})$, meaning that the right endpoint (relative to values of $G(t)$) of C_- cannot be a maximum of C. Thus the largest value of C must be the larger of $C_-(G_{\min})$ or $C_+(G_{\max})$ as required. □
