# Supplementary figures and images for: A parsimonious model of blood glucose homeostasis
Source: PLOS Digit Health. 2022 Jul 14;1(7):e0000072. doi: 10.1371/journal.pdig.0000072 (PMC9931355; doi:10.1371/journal.pdig.0000072)

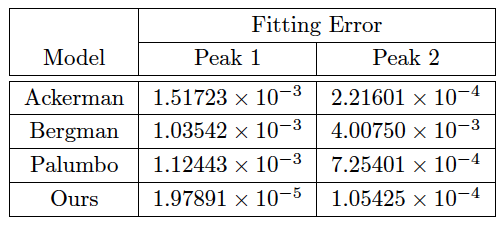

Supplement: S1 Table — The fitting error of the two sample hyperglycemic cases across different models shown in S1 Fig. The errors computed here are based on raw glucose data to accomodate for the specifications of the models compared. (TIF) [file pdig.0000072.s004.tif]

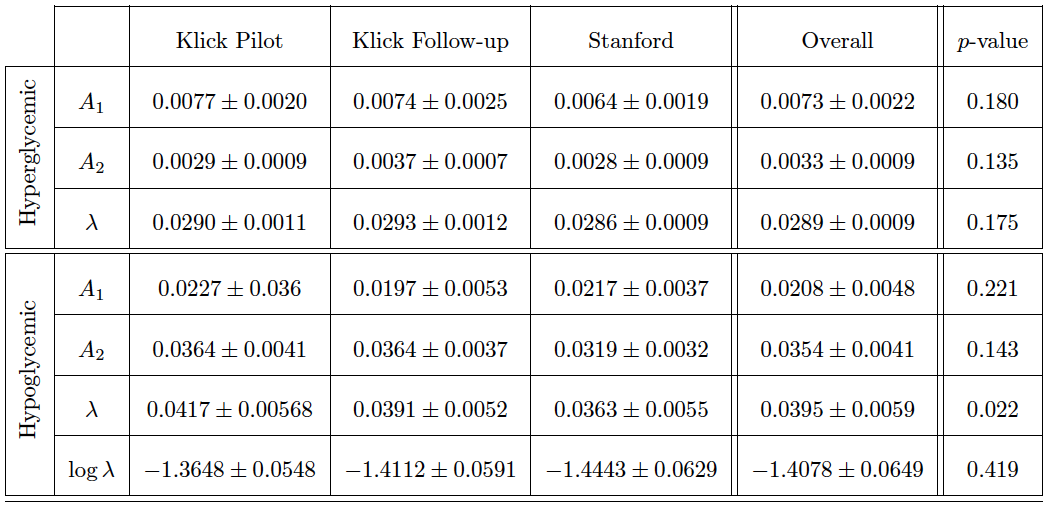

Supplement: S2 Table — Model parameter ranges for hyperglycemic and hypoglycemic cases with their respectively p-values of the Shapiro-Wilk test for normality. The null-hypothesis H0 states that the model parameters are normally distributed. The decision to reject or not reject H0 is based on a critical p-value of 0.05. The units of each parameter are listed in Table 1. (TIF) [file pdig.0000072.s005.tif]

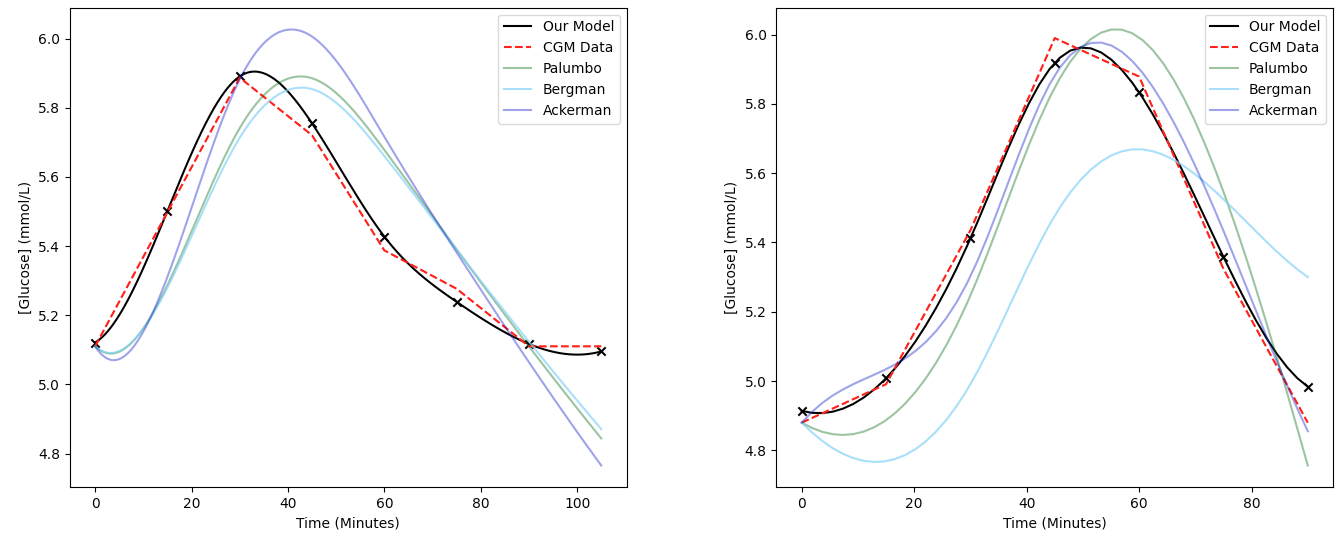

Supplement: S1 Fig — The black crosses are the model predictions at the time of each CGM measurement. The black curve is a cubic spline interpolation of the model prediction. The faded green, light blue, and purple curves are the glucose predictions based on the models proposed by Palumbo et al. [21], Bergman et al. [14], and Ackerman et al. [12], respectively. (TIF) [file pdig.0000072.s006.tif]
