# Supplementary material for: A parsimonious model of blood glucose homeostasis
Source: PLOS Digit Health. 2022 Jul 14;1(7):e0000072. doi: 10.1371/journal.pdig.0000072 (PMC9931355; doi:10.1371/journal.pdig.0000072)
Supplement: Attachment — Submitted filename: response_to_reviewers.pdf [file pdig.0000072.s013.pdf]

Acknowledgements

The authors would like to thank the reviewers for their valuable feedback. Responses to each of the reviewers' questions are provided below. The list of changes to the manuscript has been included near the end of this document.

Responses to Reviewer Questions

Reviewer 1

1. **First, as the authors indicate in the introduction section, modeling glucose metabolism is a well established topic. Beyond parsimony, what are other merits of the proposed model compared to a large number of preexisting ones? To demonstrate the merits of the proposed model the authors need to select a few "benchmark" models in this topic and compare their performance with the proposed model in terms of theoretical and data analyses.**

   Based on the authors' knowledge, the vast majority of existing models are focused on the estimation of insulin sensitivity [1, 2, 3, 4, 5]. However, in order to validate the results of these models, one requires insulin measurements, which is expensive, time consuming, and extremely inconvenient (on the participants' part) to obtain. On the contrary, our proposed model only requires participants to wear a small CGM device (roughly the size of a coin) over a two week period without any significant changes to their lifestyles. We believe that this approach could lead to better insights on the status of an individual's glucose regulation.

   We have added a small section to the manuscript (under the section "Model Fitting with CGM data") that compares the fitting results between our method and three existing methods by Ackerman [1], Bergman [2], and Palumbo [5]. The comparisons are to highlight the differences between models with different mathematical structure (linear vs nonlinear feedback, instantaneous vs delayed feedback, etc.). The representative peaks were fitted against each method and we have provided graphical comparisons of two sample representative peaks, as well as their fitting errors, in the supplementary section. These can be found under S1 Fig and S1 Table respectively in the revised manuscript. We show that our proposed method yielded better fits than all three compared models.

2. **Second, the main value of such model is to relate the inferred parameters to resilience of maintaining glucose homeostasis after external glucose sources and sinks, which is related to the normality/abnormality of glucose regulation. The data is used only for curve fitting purpose, while the content of the data is ignored. For instance, how can variations of model parameters across individuals reflect their pre-diabetic or diabetic tendency? The paper will be much more interesting if it includes some health insight derived from the data.**

   The model was validated using data obtained from healthy individuals which was a decision made by design, as one of the initial goals was to investigate any underlying structure of model parameters over individuals who are not at risk of becoming diabetic. Since the time of writing, this work has been extended to include prediabetic and diabetic individuals where we noticed that there were significant changes to parameter values as individuals became more diabetic. We surmise that these changes in parameter values reflect progressive failures of the glucose homeostatic system, and the body's inability to adapt to fluctuation levels of glucose deviation is signaled by the reduced variations among the parameter values. This work will be included and discussed in a future paper.

3. **What's the reason of choosing the feedback function phi in equation 3? Since phi is not less than $\bar{e}$, I am not clear how does the glucose level bounces back from the hypoglycemic condition.**

   The feedback function ϕ in equation 3 is to consolidate hypoglycemic and hyperglycemic feedback into a single feedback function instead of splitting them into two separate cases. During hypoglycemia, we have $e < 0$ and therefore $\phi = \bar{e}$. Hence $u < 0$ and $\phi > 0$ and so $u\phi < 0$. Because this is designed as a negative feedback system, $u\phi < 0$ corresponds to a positive change in e which raises glucose levels.

4. **Are the recovery rates from the hyperglycemic and hypoglycemic conditions similar across peaks and troughs within the same person? According to the proposed model, the variations of intra-personal rates should be smaller than those of inter-personal rates. The authors need to demonstrate this trend beyond just showing the overall fit or examples of one cycle in figures 2 and 3. Also, what are the two panels in figures 2 and 3?**

   We cannot conclude if recovery rates from hyperglycemic and hypoglycemic conditions are similar across peaks and troughs within the same person based on our model. This is because the peaks and troughs extracted throughout our method vary in amplitude and length. These variations come from a number of different sources such as different meal quantities, glucose fluctuation due to circadian rhythm, etc.. Moreover, intra-personal recovery rates are known to have significant variations even under controlled conditions. This had been previously validated by OGTT which is a standardized test of recovery from excess glucose levels, where the same test administered to the same individual at two different times had considerably different results. Because of these, the comparison of intrapersonal rates and interpersonal rates was not an assumption nor a main objective of this work, but rather, to explore and discover properties of the model parameters that can be associated and interpreted under the lens of diabetic tendencies, starting with a controlled group of healthy individuals. Since the time of writing, we have in fact extended the research to include prediabetic and diabetic individuals to identify potential digital biomarkers for robust diabetes detection, which falls outside the scope of this manuscript.

   The panels in figures 2 and 3 are sample representative peaks and troughs extracted from our peak/trough selection process, then fitted to our model. The captions and descriptions of these figures have been modified for better clarity.

Reviewer 2

1. **How exactly is the "OGTT episode" extracted from the data (see comments below also)?**

   We did not extract OGTT episodes from data because a primary goal is to analyze CGM data collected during the participants' daily lives as opposed to a controlled test environment. Measurements of OGTT (and other metrics) were obtained after the CGM device was removed (at the end of CGM data collection). OGTT measurements are simply used to provide an idea of the true diabetic statuses of participants and it is not used to perform any of the analysis described by our method.

2. **How reproducible is the fitting procedure, that is, in what fraction of the CGMs can this be carried out without human assistance?**

   During the early stages of this project, there were a number of experimentations needed before we arrived at the current version of the model. The main steps that required many experimentation were the peak selection procedure, baseline selection of peaks/troughs, and initial conditions during the data fitting process. After numerous trials, we noticed that there were little difference from a qualitative standpoint, and thus it was preferable to keep the peak/trough/baseline selection procedure simple.

   At its current state, the fitting procedure in its entirety is automatic aside from minor data preprocessing of CGM data. The fitting procedure takes in CGM data in csv (comma separated values) format where each entry contains the time of measurement (in minutes) relative to the time of the first data sample, and glucose concentration reading (in mmol/L) from the CGM device. The rest of the procedure (peaks/troughs selection, parameter fitting, post-fitting analysis) are fully automated and require no manual intervention.

3. **I recommend changing the notation, e, for excess glucose. It is confusing to read it together with the "exp", say in Eq. 2.**

   Thank you for the feedback. We agree with your assessment regarding the confusing nature of the variable names. To better improve clarity, all mentions of e in the manuscript will refer to the time-dependent glucose deviation function $e(t)$, while any mathematical statements involving the constant e is replaced by an equivalent statement involving "\exp".

4. **At the bottom of page 4. where the datasets are described, please add a short description to place the data in context. For instance, are these (all) type 2 or type 1 diabetic patients?**

   The recruitment of non-diabetic individuals was a decision by design (see lines 145-146), as one of the initial primary goals is to investigate any underlying structure of model parameters over individuals who are not at risk of becoming diabetic. Since the time of writing, this work has been extended to include prediabetic and diabetic individuals where we noticed that an increased diabetic tendencies lead to significant changes in parameter values. We surmise that these changes in parameter values reflect progressive failures of the glucose homeostatic system, and the body's inability to adapt to fluctuation levels of glucose deviation is signaled by the reduced variations among the parameter values.

5. **Lines 230-end of Section: Please use an example to demonstrate your methodology. The reason is that it is not completely obvious to what extent this method would succeed? For instance, how do you choose $\bar{e}$? Is it picked ahead of time, at a specific time in the morning, or by averaging the trace over a day etc.?**

   The model takes CGM data which is smoothed to suppress instrumentation noise. Potential peaks/troughs are then extracted from the full CGM data, where each peak/trough is then fitted to the proposed model. This generates a set of model parameter values for each extracted peak/trough. The choice of $\bar{e}$ is specific to each peak/trough by defining $\bar{e}$ as the minimum/maximum of the peak/trough. Therefore the values of $\bar{e}$ are not based on a specific time of the day, but rather on the individuals' activities that cause glycemic excursions (both positive and negative).

6. **The mathematical analysis (theorems/proofs) is based on fairly standard techniques. It might be better to place this in an Appendix to improve the readability of the main text.**

   We believe that the theorems and corollaries in the main body of the paper are statements of immediate results from the proposed model. The derivations of each result (e.g. proofs) are left out of the main text and included in supporting information under the appropriate headings.

List of Changes

The revised manuscript containing the tracked changes is located in `parsimonious_glucose_model_submission_revised1_tracked.pdf`. Removed text is crossed out and highlighted in red, and additions are highlighted in blue. The following is the list of changes in the revised manuscript since the initial submission:

- (Eq 2 - Page 5) Replaced $e^{-\lambda(t-\tau)}$ with $\exp(-\lambda(t-\tau))$ to better distinguish the difference between the constant e and the glucose deviation function $e(t)$.
- (Lines 243-244 - Page 7) Correction to the explanation of the error function.
- (Line 283 - Page 8) Extended theorem 2 to show that no periodic solutions exist.
- (Lines 312-328, 330-332 - Page 9-10) Added a description and discussion of the comparisons between our proposed model and existing models by Bergman [2], Palumbo [5], and Ackerman [1].
- (Fig 2, Fig 3 - Pages 10) Included an additional sentence to clarify the description of the associated plots.
- (S2 Theorem, Page 13) Extended the proof of S2 Theorem to show that there is no periodic orbits inside the trapping region.
- (S1 Table, Page 14) Included a table of comparison, and description, of fitting errors between our proposed model and existing models by Bergman [2], Palumbo [5], and Ackerman [1].
- (S2 Table, Page 14) Table removed from the base manuscript to be included as an external attachment.
- (S1 Fig, Page 15) Included figure comparing model prediction results between our proposed model and existing models by Bergman [2], Palumbo [5], and Ackerman [1].

- (Various pages) Minor updates to labelling and references due to the additional tables and figures added to the revised manuscript.

References

[1] Ackerman E, Gatewood LC, Rosevear JW, Molnar GD. Model studies of blood-glucose regulation. Bull Math Biophys. 1965;27:21–73.

[2] Bergman RN, Ider YZ, Bowden CR, Cobelli C. Quantitative estimation of insulin sensitivity. Am J Physiol Endocrinol Metab. 1979;236:E66–77.

[3] Saunders PT, Koeslag JH, Wessels JA. Integral Reign Control in Physiology. J Theor Biol. 1998;194:163–173.

[4] Steil GM, Rebrin K, Janowski R, Darwin C, Saad MF. Modeling β-cell insulin secretion-implications for closed-loop glucose homeostasis. Diabetes Technol Ther. 2003;5:953–964.

[5] Palumbo P, Gaetano AD. State feedback control of the glucose-insulin system. In: Aletti G, Burger M, Micheletti A, Morale D, editors. Math Everywhere: Deterministic and Stochastic Modelling in Biomedicine Economics and Industry. Springer; 2007. p. 241–252.
